# Prevalence and Risk Factors of Myopia in Spain

**DOI:** 10.1155/2019/3419576

**Published:** 2019-08-18

**Authors:** Cristina C. Alvarez-Peregrina, Miguel Angel M. A. Sanchez-Tena, Clara C. Martinez-Perez, Cesar C. Villa-Collar

**Affiliations:** Faculty of Biomedical and Health Sciences, Universidad Europea de Madrid, Madrid 20822, Spain

## Abstract

**Objective:**

To analyse the prevalence of myopia among a sample of more than 6000 children in Spain as well as to determine the impact of risk factors in its progression.

**Methodology:**

A total of 6,152 children aged from 5 to 7 were examined. The participants underwent an eye examination that included visual acuity, refraction without cycloplegia, and tests of accommodative and binocular function. In addition, a questionnaire regarding their lifestyle, family history, and geographical data was carried out. Finally, data were analysed using the SPSS version 25 program.

**Results:**

The prevalence of myopia in the sample of children studied has increased from 17% in 2016 to 20% in 2017. Likewise, the number of children with high myopia has also increased, from 1.7% in 2016 to 3.6% in 2017. 43.3% of the participants spent more than 3 hours a day doing near activities, and 48.9% of this group spent more than 50% of this time using electronic devices. In addition, only 9.7% spent more than 2.5 hours outdoors each day.

**Conclusion:**

Myopia prevalence appears to be increasing in Spain. Lifestyle factors appear to be increasing the risk of myopia.

## 1. Introduction

Uncorrected refractive errors are one of the main public health problems throughout the world, regardless of age, sex, and race [1]. As a result, it is expected that by 2060, there will have been a 26% increase in the number of children with visual disability, which will have a negative effect on their educational and psychosocial development [2, 3].

In recent years, there has been a significant increase in the number of cases of myopia globally, and it has become an epidemiological problem [4]. Between 1993 and 2016, the prevalence rate increased from 10.4% to 34.2%, respectively [5]. Short-term estimates indicate that in 2050, 49.8% of all people will be myopic [6].

The prevalence of myopia varies on a geographical basis; it is more prevalent in Asia (70–90%) [7], whereas the figures appear to be lower than in Europe, Australia, and USA [8]. Regarding this, recent studies have determined a higher myopia rate among the children examined in Singapore (62%) and China (49.7%) in comparison with those examined in the USA (20%) and Australia (11.9%) [6, 9]. Additionally, high myopia could be associated with multiple pathologies including retinal detachment, macular degeneration, cataracts, or glaucoma [10]. However, there are no current data about the myopia prevalence in Spain since 2000, when myopia incidence in children from 3 to 8 years old was 2.5% [11].

Nowadays, there is enough evidence on the influence of near activities (reading, writing, watching TV, etc.), in the development of myopia. The hypermetropic peripheral blur in the retina leads to an increase in the axial length of the eye, therefore accelerating its progression [12].

Genetics also plays an important role, so the risk of suffering myopia increases depending on the number of parents with myopia [13].

Recent studies suggest that time outdoors has a protective effect on the appearance of myopia, but it does not stop its progression [14].

An important point when we look at prevalence figures is to know the procedure to measure myopia. The recent report published by the IMI group of experts—*Defining and Classifying Myopia Report*—defines myopia by refraction “when ocular accommodation is relaxed. These definitions avoid the requirement for objective refraction so as to be independent of technique, but by making reference to relaxation of accommodation are compatible with both cycloplegic and standard clinical subjective techniques” [15]. Although cycloplegic refraction is the gold standard, limitations in the use of some drugs in some countries make important having other alternatives to measure myopia, like objective refraction by noncycloplegic retinoscopy.

If we assess the economic impact which is associated with myopia, a study carried out in 2013 estimated a total cost in the whole population of Singapore of 755 million US dollars per year [16].

Therefore, due to the lack of studies of myopia prevalence in Spain and the need to know which associate factors can help to prevent this epidemiologic problem, the authors carried out this study. We analysed myopia prevalence among children from 5 to 7 years old and the influence of lifestyle and genetics in the figures.

## 2. Methods

### 2.1. Data Collection and Inclusion Criteria

A cross-sectional study to estimate myopia prevalence in a sample of children in Spain has been carried out.

Data were collected by convenience sampling from the 2016 and 2017 “School campaign in favor of children's visual health” that is taken every year in Spain. The school campaign is targeted to all schools, so all participants between 5 and 7 years of age that participated were included in the study. The school campaign supplies a free spectacle to those who need them, funded by the Fundación Alain Afflelou.

### 2.2. Examination

Parents of all of the children that participate in this research signed the informed consent form and underwent an optometric test, which consisted of a questionnaire and an assessment of the refractive and binocular conditions:

- Questionnaire: it was divided into several sections and included questions about their *demographic data* (city of residence, age, sex, and nationality), their *lifestyle and family ocular history* (extracurricular activities and number of hours/weeks spent doing these activities, time spent using electronic devices, and genetics), and *anamnesis* (symptoms, main complaint, diagnosis or previous ocular treatment, medication and systemic diseases, and date of last checkup).
- Optometric test: the standard procedure was as follows:
  1. Best-corrected and uncorrected visual acuity.
  2. Objective refraction: non cycloplegic retinoscopy. The authors have estimated differences of ±0.5*D* in the SE when comparing noncycloplegic retinoscopy versus cycloplegic refraction [17].
  3. Subjective refraction.
  4. Binocular vision and accommodative tests: cover-uncover, alternating cover test, ocular motility, Hirschberg test, Worth test, near point of convergence, accommodation range, stereopsis, and colour vision.
  5. Finally, the anterior segment was checked (eyelid, eyelashes, palpebral margin, corneal, conjunctive, and crystalline) using a slit lamp.

### 2.3. Variable Description

In order to determine the refractive status of the children, and in accordance with other research, the criteria for the spherical equivalent (SE) were as follows: hyperopia (S.E. > +0.50), myopia (S.E. < −0.50), or emmetropia (−0.50 < S.E. > +0.50) [2, 15]. SE was defined as *sphere* + *cylinder*/2.

Within the myopic group, a subdivision of myopia was carried out, based on the *American Academy of Optometry'*s classifications [18] as low (−0.50 < S.E. > −3), medium (−3 < S.E. > −6), and high (S.E. > −6).

To calculate the number of hours that children spend in near activities, using electronic devices and outdoors, and to get the genetic risks, several variables were taken based on the *Clinical Myopia Profile* [19]. Therefore, according to this study, we estimated the risk of suffering myopia in high, medium, or low, taking into consideration the criteria shown in Table 1.

### 2.4. Statistical Analysis

The data analysis was carried out using the SPSS 25.0 program (SPSS Inc., Chicago, Illinois). To establish the parametric distribution of the variables, the Kolmogorov–Smirnov test was used, resulting in a nonparametric distribution. Therefore, the variables were analysed using the Kruskal–Wallis test. The prevalence was calculated with 95% confidence interval. To assess the statistical significance, we considered a cutoff point *p* ≥ 0.05.

## 3. Results

The checkouts were carried out in September 2016 and September 2017. A total of 6152 children were examined (4159 in 2016 and 1993 in 2017). A total of 711 children were excluded: 210 participants did not fulfill the inclusion criteria (younger than 5 or older than 7 years old) and 501 forms were incomplete as the optometrist didn't follow method properly. The average age was 6.17 ± 0.77 years (2016: 6.16 ± 0.77 years old; 2017: 6.19 ± 0.78 years old). In terms of gender, 55% were male and 45% were female (2016: 56.3% male; 43.7% female; 2017: 52.5% male; 47.5% female).  Table 2 shows the percentage of participants from the different autonomous communities across Spain by age and sex.

**Figure 1:**
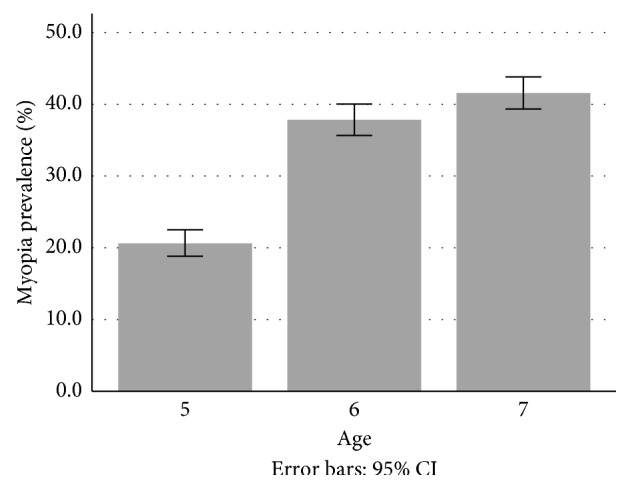
Myopia prevalence according to age.

Figures of myopia prevalence in children aged between 5 and 7 years increased from 16.8% in 2016 to 19.1% in 2017 (OR: 1.19; IC: 1.16–1.22; *p* ≤ 0.001). Likewise, the percentage of cases of myopia in female increased by 1.6% (16.5% in 2016, *p*=0.127; 18.1% in 2017, *p*=0.294; average: 17.25 ± 1.2%) and 3% in male (17% in 2016, *p*=0.216; 20% in 2017, *p*=1; average = 18.55 ± 2.05%). Therefore, no statistically significant differences were found between the risk of suffering from myopia and gender (*p*=0.134). With regards to age, Figure 1 shows how the prevalence of myopia increases progressively with age (*p* ≤ 0.001).

Table 3 shows myopia prevalence by gender and place in 2016 and 2017.

Out of all of the participants with myopia, in 2016, 90.1% had low myopia, 8.2% had medium myopia, and 1.7% had high myopia. On the other hand, in 2017, the percentage of children with low myopia was 89.1%, with a slight increase in the moderate myopia rates (9%) and the high myopia rates (1.9%). Likewise, there was an increase in the number of individuals who used glasses, from 70.6% in 2016 to 81.5% in 2017. In relation to this and with regards to the prevalence of myopia in the different autonomous communities, statistically significant differences have been found (*p* ≤ 0.001).

The spheric myopic equivalence values according to age, sex, and autonomous community in 2016 and 2017 can be observed in Table 4.

### 3.1. Risk Factors

To assess the number of hours in which participants perform near activities, three groups were established: low (between 0 and 2 hours), moderate (between 2 and 3 hours), and high (more than 3 hours). To determine the time spent using electronic devices, three subgroups were established, according to whether they spend <25%, between 25% and 50%, or more than 50% of the time in near activities.

In both 2016 and 2017, 45.5% and 39.7% of the children, respectively, spent a lot of time carrying out near activities. However, 36.1% (35.9% in 2016 and 36.3% in 2017) spent few hours and 21.2% (19.3% in 2016 and 24.1% in 2017) spent a moderate amount of time.

With regards to the use of electronic devices, 48.3% of the children (57.9% in 2016 and 33.1% in 2017) used them >50% of the time in near activities. Only 26.2% (21.9% in 2016 and 32.9% in 2017) used them <25% of the time and 25.6% (20.2% in 2016 and 34% in 2017) between 25% and 50%.

**Figure 2:**
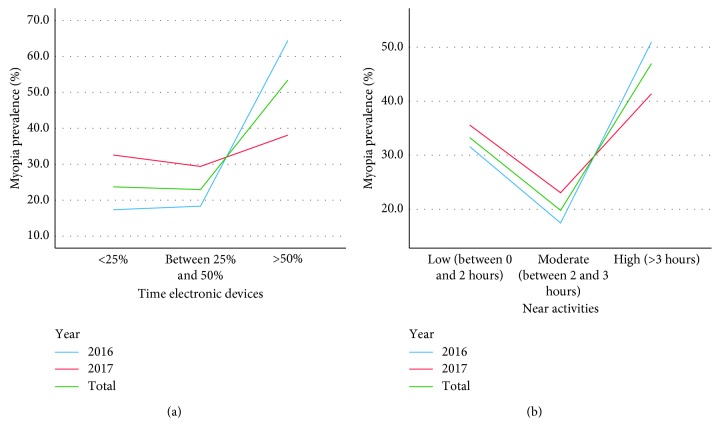
Prevalence of myopia according to (a) the use of electronic devices and (b) the time spent performing activities in near vision.

Figure 2 shows that the more time spent performing near activities and using a phone, tablet, or videogames, the higher the prevalence of myopia (*p* < 0.05).

On the other hand, a moderate correlation was found between the spherical equivalent value with regards to the time spent in near activities and using electronic devices (*p* < 0.05).

**Figure 3:**
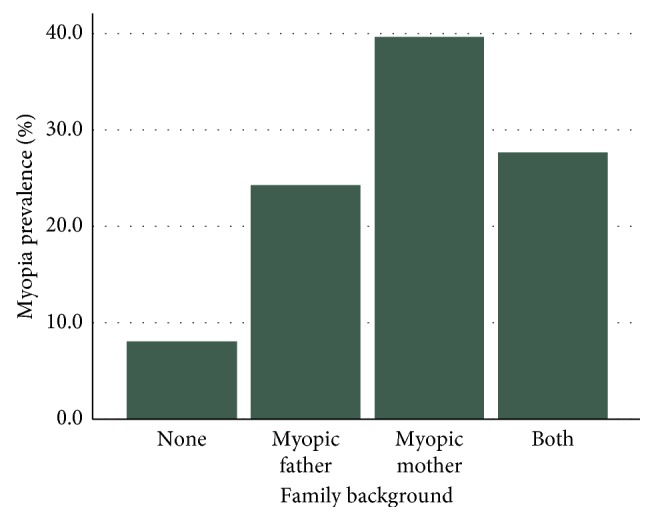
Percentage of refractive condition of children in relation with their family history.

With regards to the predisposition, as shown in Figure 3, a significant association has been found between the presence of myopia in one or both parents and the refractive condition of the children (*p*=0.013). Therefore, the risk of having myopia increases from 9.7% if neither parent is myopic to 28.3%, if both are, respectively.

### 3.2. Prevention Factors

Each child was allocated to a group depending on the hours he spent outdoors each day: low (between 0 and 1.6 hours), moderate (between 1.6 and 2.7 hours), and high (>2.7 hours). 80.7% of the participants spent short time outdoors, while only a 9.9% of the children spent a moderate amount of time, and 9.4% of children spent long time outdoors, respectively.

However, in this study, we did not obtain statistically significant differences between the prevalence of myopia and the time they spend outdoors (*p*=0.961).

## 4. Discussion

According to the WHO, myopia is considered as one of the main public health problems worldwide [20]. Our study included a group of children between 5 and 7 years of age, of which 18% were myopic in 2016 and 2017. Therefore, it has been concluded that figures of myopia prevalence in our sample of children in Spain are similar to that of Australia (14.02%) [21], Central Asia (17%), Andean Latin America (20.5%), and Tropical Latin America (14.5%) [6]. Contrasting, figures of prevalence are higher in Pakistan (36.5%) [22] and in Saudi Arabia (53.71%) [23].

Regarding gender, we did not find any significant differences in the prevalence of myopia. These results agree with those obtained by Uchenna et al. [24] and COMET [25], showing that there is no connection between sex and myopia and that figures can vary along time. However, there are studies, like the ones carried out in China [26, 27] and Saudi Arabia [28], that show higher figures of myopia prevalence in female than in male.

According to other studies, the prevalence of myopia increases with age. Thus, in 2016, Ma et al. [29] indicated an increase of 50.4% in children from 3 to 10 years old. When comparing the SE value of our research with the one carried out by Pi et al. [30] in 2010, a tendency of myopisation is observed, going from +1.25*D* in 2010 versus +0.78*D*, found in our study, in 2017. Likewise, similar studies show an increase in S.E. value of −0.27*D* per year, in 50% of the children [31].

With regards to lifestyle, the latest reviews indicate that children spend on average 4.8 ± 1.6 hours each day doing near activities. Likewise, it was shown that male spend more time doing near activities than female (4.9 ± 1.7 vs 4.6 ± 1.5) [32]. In 2006, Khader et al., proved that children with myopia spend around 0.95 hours/day in front of a computer, as opposed to the 0.69 hours/day spent by nonmyopic children [33]. These results agree with the ones obtained in our study in Spain. On the other hand, Lu et al. [34], Rose et al. [35], and Lin et al. [36] have pointed out that near activities are not a risk factor in the development of myopia.

With regards to the time spent outdoors, we found that most children spend between 0 and 1.6 hours outdoors. Similar results were obtained in Sydney in 2008, where children spend around 2.3 hours/day outdoors [37]. This difference could be due to the greater use of electronic devices nowadays and the geographical location.

There are a lot of studies that look for relations between spending outdoors time and myopia. Jin et al. [38] found the less figures of myopia, by means of pupil constriction and the release of dopamine, the greater the exposure to sunlight. However, we did not find a connection between the time spent outdoors and prevalence of myopia. This leads us to believe that in Spain, no association has been found due to the lack of children in our sample who spend more than 2.5 hours per day exposed to sunlight; therefore, it would be interesting to confirm these results through future research.

With regards to the limitations of our study, it is important to highlight the low number of participants aged 5 years (23%), in comparison with 37% of 6-year-old children and 40% of 7-year-old children, respectively. It is also important to say that centres from Balearic Islands, Melilla, and La Rioja did not participate in the 2017 collection, so comparison between 2016 and 2017 has not been included for these autonomous communities in Tables 3 and 4. In addition, only noncycloplegic refraction has been taken in this study, so it must be taken into consideration when compared to other studies. Similar studies have found that the difference between noncycloplegic and cycloplegic refraction is 0.95*D* in young children [39]. Finally, it should also be noted that the campaign offered a free spectacle to children that needed, so it could suppose a bias in the study.

## 5. Conclusion

Myopia prevalence appears to be increasing in Spain. Lifestyle factors appear to be increasing the risk of myopia.

## Figures and Tables

**Table 1:** Factors that affect the risk of suffering from myopia.

	High risk	Medium risk	Low risk
Time spent outdoors (with sun light)	Short time (between 0 and 1.6 hours)	Moderate time (between 1.6 and 2.7 hours)	Long time (>2.7 hours)
Time spent doing near activities (excluding school time)	Long time (>3 hours)	Moderate time (between 2 and 3 hours)	Short time (between 0 and 2 hours)
Family history	Both parents suffer from myopia	One of the parents suffer from myopia	Any of parents suffer from myopia

Source: [19].

**Table 2:** Participants from the different autonomous community by age and gender.

	Male				Female			
	5 years	6 years	7 years	Total	5 years	6 years	7 AÑOS	Total
	*N* (%)	*N* (%)	*N* (%)	*N* (%)	*N* (%)	*N* (%)	*N* (%)	*N* (%)
Basque country	32 (4.5%)	59 (5.4%)	50 (4.2%)	141 (4.7%)	33 (6.2%)	50 (5.4%)	38 (3.8%)	121 (4.9%)
Andalusia	92 (12.9%)	151 (13.9%)	189 (15.9%)	432 (14.5%)	68 (12.8%)	104 (11.2%)	119 (12%)	291 (11.9%)
Valencian Community	36 (5.1%)	68 (6.2%)	91 (7.7%)	195 (6.5%)	23 (4.3%)	6 (6.8%)	59 (6%)	145 (5.9%)
Asturias	0 (0%)	0 (0%)	1 (0.1%)	1 (0%)	—	—	—	—
Catalonia	65 (9.1%)	116 (10.7%)	116 (9.8%)	297 (9.9%)	62 (11.6%)	106 (11.4%)	110 (11.1%)	278 (11.3%)
Castile and Leon	113 (15.9%)	203 (18.6%)	202 (17%)	518 (17.3%)	86 (16.1%)	171 (18.4%)	168 (17%)	425 (17.3%)
Galicia	43 (6%)	52 (4.8%)	58 (4.9%)	153 (5.1%)	30 (5.6%)	37 (4%)	58 (5.9%)	125 (5.1%)
Community of Madrid	164 (23%)	218 (19.9%)	219 (18.5%)	601 (20.1%)	104 (19.5%)	167 (18%)	163 (16.4%)	434 (17.7%)
Aragon	53 (7.4%)	61 (5.6%)	79 (6.7%)	193 (6.5%)	36 (6.8%)	63 (6.8%)	67 (6.8%)	166 (6.8%)
Cantabria	18 (2.5%)	28 (2.6%)	23 (1.9%)	69 (2.3%)	11 (2.1%)	21 (2.3%)	18 (1.8%)	50 (2%)
Navarra	23 (3.2%)	28 (2.6%)	28 (2.4%)	79 (2.6%)	14 (2.6%)	28 (3%)	37 (3.7%)	79 (3.2%)
Extremadura	32 (4.5%)	46 (4.2%)	46 (3.9%)	124 (4.1%)	32 (6%)	56 (6%)	65 (6.5%)	153 (6.2%)
Murcia	0 (0%)	2 (0.2%)	1 (0.1%)	3 (0.1%)	1 (0.2%)	0 (0%)	2 (0.2%)	3 (0.1%)
Castile-la Mancha	24 (3.4%)	42 (3.8%)	55 (4.6%)	121 (4%)	17 (3.2%)	39 (4.2%)	52 (5.3%)	108 (4.4%)
Balearic Islands	9 (1.3%)	7 (0.6%)	7 (0.6%)	23 (0.8%)	7 (1.3%)	16 (1.7%)	18 (1.8%)	41 (1.7%)
Melilla	2 (0.3%)	5 (0.5%)	0 (0%)	7 (0.2%)	3 (0.6%)	2 (0.2%)	0 (0%)	5 (0.2%)
La Rioja	6 (0.8%)	6 (0.5%)	21 (1.8%)	33 (1.1%)	6 (1.1%)	4 (0.4%)	17 (1.7%)	27 (1.1%)
Total	712 (100%)	1093 (100%)	1186 (100%)	2991 (100%)	533 (100%)	926 (100%)	990 (100%)	2450 (100%)

**Table 3:** Myopia prevalence by gender and place in 2016 and 2017.

	Gender/autonomous community	2016				2017			
		5 years (%)	6 years (%)	7 years (%)	Total (%)	5 years (%)	6 years (%)	7 years (%)	Total (%)
Medium age	Female	46.4	42.1	41.7	42.7	46.5	47.8	42.2	44.8
	Male	53.4	57.9	58.3	57.3	53.5	52.2	57.8	55.2
6.09 ± 0.76 years	Basque country	4.8	5.5	4.6	5	—	8.2	4	4.7
6.29 ± 0.79 years	Andalusia	16.8	20.5	18.7	19	6.3	6.5	13.3	9.8
6.27 ± 0.74 years	Valencian community	8.2	6	6.1	6.4	3.9	4.5	5.7	5
6.17 ± 0.76 years	Catalonia	12	7.6	9.4	9.3	17.3	14.7	8.2	12
6.18 ± 0.76 years	Castile and Leon	14.9	20.5	18.5	18.6	16.5	19.2	21.8	20
6.15 ± 0.81 years	Galicia	2.9	4.3	2	2.9	3.1	5.3	5.9	5.2
6.11 ± 0.79 years	Community of Madrid	17.8	17.6	18.4	18	31.5	23.7	23.2	24.8
6.16 ± 0.79 years	Aragon	3.4	2.1	2.9	2.7	9.4	9	10.8	9.9
6.10 ± 0.76 years	Cantabria	2.9	0.2	1.2	1.2	3.1	1.2	—	1
6.18 ± 0.86 years	Navarra	1	0.5	0.7	0.7	6.3	3.3	4.2	4.3
6.17 ± 0.78 years	Extremadura	3.8	6.2	5.5	5.5	1.6	2.9	1.4	1.9
6.29 ± 0.75 years	Castile-la Mancha	7.2	6.9	7	7	0.8	1.6	1.4	1.4

**Table 4:** Myopic spherical equivalence according to age, sex, and autonomous community.

		2016			2017		
		5 years	6 years	7 years	5 years	6 years	7 years
Sex	Female	−1.55 ± 0.97	−1.55 ± 1.12	−1.66 ± 1.21	−1.55 ± 0.99	−1.27 ± 0.80	−1.55 ± 1.09
	Male	−1.55 ± 1.65	−1.59 ± 1.34	−1.66 ± 1.58	−2.51 ± 3.27	−1.68 ± 1.84	−1.73 ± 1.41
Autonomous community	Basque Country	−1.38 ± 1.11	−1.23 ± 0.81	−1.38 ± 0.95	—	−1.36 ± 0.48	−1.84 ± 2.20
	Andalusia	−1.86 ± 2.11	−1.82 ± 1.63	−1.62 ± 1.23	−1.51 ± 0.57	−1.32 ± 0.81	−1.70 ± 1.23
	Valencian Community	−1.32 ± 0.89	−1.78 ± 1.97	−1.25 ± 0.78	−3.65 ± 1.43	−1.48 ± 0.92	−1.35 ± 1.01
	Catalonia	−0.99 ± 0.82	−1.38 ± 0.98	−1.47 ± 1.06	−2.16 ± 2.68	−2.30 ± 2.83	−1.34 ± 9.02
	Castile and Leon	−1.20 ± 0.52	−1.37 ± 0.52	−1.55 ± 1.22	−1.34 ± 0.91	−1.12 ± 0.76	−1.77 ± 1.16
	Galicia	−1.10 ± 0.39	−1.67 ± 1.43	−1.89 ± 0.58	0.35 ± 3.02	−1.55 ± 0.90	−1.70 ± 2.02
	Community of Madrid	−1.05 ± 0.64	−1.47 ± 1.13	−2.14 ± 2.29	−2.26 ± 3.23	−1.39 ± 1.44	−1.51 ± 1.28
	Aragon	−0.91 ± 0.39	−2.80 ± 1.30	−1.57 ± 0.68	−3.20 ± 2.75	−1.37 ± 0.77	−2.08 ± 1.28
	Cantabria	−1.46 ± 0.67	−0.50 ± —	−2.18 ± 2.45	−3.19 ± 1.99	−1.50 ± 0.90	—
	Navarra	−7.75 ± 0.00	−0.50 ± 0.00	−1.28 ± 0.47	−1.62 ± 1.66	−1.22 ± 0.61	−1.42 ± 0.62
	Extremadura	−1.12 ± 0.63	−1.06 ± 0.80	−1.39 ± 1.03	−1.94 ± 0.88	−1.73 ± 0.77	−1.15 ± 0.36
	Castile-la Mancha	−1.41 ± 0.79	−1.41 ± 0.91	−1.46 ± 1.14	−2.00 ± —	−1.56 ± 0.33	−2.15 ± 1.23

## Data Availability

The data used to support the findings of this study are available from the corresponding author upon request.
